# Supplementary material for: Mindfulness Practice versus Physical Exercise in Enhancing Vitality
Source: Int J Environ Res Public Health. 2023 Jan 31;20(3):2537. doi: 10.3390/ijerph20032537 (PMC9916355; doi:10.3390/ijerph20032537)
Supplement: Supplementary file 1 [file ijerph-20-02537-s001.zip › ijerph-2054092-supplementary.pdf]

Supplementary materials for
Mindfulness practice versus physical exercise in enhancing vitality

A Brief Introduction of Research Article
Accepted by Chinese Social Psychological Review (in Chinese)

The Theoretical Construction and Measurement of Vitality in the Chinese Cultural Context

Wei Yan[1], Peng Zhang[1], Zhongxin Jiang[2], Guanmin Liu[1], Kaiping Peng[1]
[1] Department of Psychology, School of Social Sciences, Tsinghua University, Beijing, China
[2] Department of Applied Psychology, Chinese University of Hong Kong, Shenzhen, China

The seminal work of Ryan and Frederick (1997) introduced the construct of vitality as one's positive feeling of physical and mental energy available to oneself, which could be regulated and harnessed to conduct purposive actions. Based on this definition, the Subjective Vitality Scale (SVS; Ryan & Frederick, 1997) was developed to measure the construct. Nevertheless, this line of research has been largely conducted in Western cultural context. Little is known about how Eastern Asians, especially Chinese, view vitality. Though SVS demonstrates good reliability and validity, we were interested in developing an alternative vitality scale that reflects Chinese people's understanding of the vitality construct. To develop the scale, three studies were conducted.

In Study 1, 135 Chinese participants (aged 31-55) were recruited and interviewed about their understanding of vitality. From the coding of interview transcripts, four factors of vitality were obtained: *Energy* (i.e., the feeling of physical and mental energy available to self), *tenacity* (i.e., volitional and behavioral perseverance), *serenity* (i.e., balanced utilization of energy available to self) and *acuteness* (i.e., alertness to inner and outer changes). The results suggest that Chinese consider vitality as a multi-facet construct.

In Study 2, we initially generated a total of 30 items (in Chinese) that were in accord with the concept of each vitality factor introduced above (i.e., energy: 7 items; tenacity: 8 items; serenity: 8 items; acuteness: 7 items). The 30-item scale was administrated to a sample of 502 Chinese adults (aged 18-52), with the instruction to score their agreement or disagreement with each item using a 7-point Likert scale (1 = *Strongly Disagree*, 7 = *Strongly Agree*). The total score represents the level of vitality in general, and the factor scores represent the levels of each vitality factor. The initial item analyses resulted in deletion of 9 items from the scale, which have poor discrimination (i.e., the item score showed no significant difference between groups with top 27% and bottom 27% total score) or a loading of less than 0.4 on each factor with an eigenvalue greater than 1.

Afterwards, the 21-item scale and SVS were administrated to a sample of 672 Chinese adults (aged

18-67). All 21 items showed good discrimination (*p*s < 0.05; See Table S1). The whole sample of 672 was randomly divided into two sub-samples of 336 to perform exploratory factor analysis (EFA) and confirmatory factor analysis (CFA), respectively. The initial EFA extracted four factors with an eigenvalue greater than 1, which explained 61.13% of total variance (see Table S2). Four items with a loading of less than 0.4 on all the four factors were further deleted from the scale. The subsequent EFA with the remaining 17 items extracted four factors with an eigenvalue greater than 1, which explained 69.66% of total variance (see Table S3). Further, the CFA examined a four-factor model on the 17-item scale (see Figure 1) and showed a good fit of model (i.e., $\chi^2 = 284.22$, $df = 113$, $\chi^2/df = 2.52 < 3.00$, TLI = 0.93 > 0.90, CFI = 0.94 > 0.90, SRMR = 0.05 < 1.00, RMSEA = 0.07 < 0.08).

With these results, a final 17-item version of the scale was retained and named as Four-Factor Vitality Scale (FFVS: Energy-5 items, tenacity-4 items, serenity-4 items, acuteness-4 items; see attachment for the scale items). The FFVS has good internal consistency, with Cronbach's α of 0.90 (total score), 0.92 (energy sub-scale), 0.84 (tenacity sub-scale), 0.81 (serenity sub-scale), and 0.78 (acuteness sub-scale). In addition, the total score of FFVS was strongly correlated with the SVS score ($r = 0.75$; see Table S4). The SVS was particularly highly correlated with the energy factor ($r = 0.82$), which suggests that the energy factor in FFVS can be considered as identical to subjective vitality. On the other hand, the SVS was correlated in smaller magnitude with the factors of tenacity ($r = 0.62$), serenity ($r = 0.46$) and acuteness ($r = 0.24$).

Lastly, the FFVS was administrated to a sample of 65 Chinese adults (aged 24-61) twice with an interval of 14 days, so as to examine its retest reliability. The results showed high correlations between scores measured at the two time points, with coefficients of 0.82 (total score), 0.90 (energy sub-scale), 0.78 (tenacity sub-scale), 0.85 (serenity sub-scale), and 0.76 (acuteness sub-scale), suggesting a good retest reliability of FFVS.

Study 3 was conducted to further examine the criterion validity of FFVS. In the study, a sample of 291 Chinese adults (aged 18-27) was recruited and completed FFVS and the measure of courage (Values in Action Inventory of Strengths, VIA-IS; Peterson & Seligman, 2004). Another sample of 2020 Chinese adults (aged 20-60) was recruited and completed FFVS and the measures of life satisfaction (The Satisfaction With Life Scale, SWLS; Diener et al., 1985), positive mental health (The Short Warwick-Edinburgh Mental Well-being Scale, SWEMWBS; Stewart-Brown et al., 2009), resilience (Connor-Davidson Resilience Scale-2, CD-RISC-2; Vaishnavi et al., 2007), depression (The Patient Health Qustionnarie-2, PHQ-2; Kroenke et al., 2003) and loneliness (UCLA Loneliness Scale-4, UCLA-4; Russell et al., 1980). As can be seen in Table S5 and S6, the correlations between FFVS and these measures showed that, (a) the FFVS and its factors were positively correlated with courage and its factors, and (b) the FFVS and its factors were positively correlated with life satisfaction, positive mental, and resilience, and with depression and loneliness. These findings suggested a good criterion validity of FFVS.

Conclusion:

Through three studies, we provided evidence that (1) Chinese people view the construct of vitality with a four-factor model, which includes the factors of energy that commonly shared by both Chinese and Western populations, and the factors of tenacity, serenity and acuteness that are more specific to Chinese populations; (2) the Four-Factor Vitality Scale showed good reliability and validity, which can serve as a valid and reliable tool to measure vitality among Chinese populations.

Table S1

The discrimination analysis for the 21 items by the comparisons between high score group and low score group

Item	Average item score	Standard deviation	Average item score of high- (top 27%) score group	Average item score of low- (bottom 27%) score group	*t*(360)
H1	5.35	1.23	6.29	4.39	17.23
H2	5.73	1.12	6.57	4.68	18.67
H3	5.45	1.28	6.46	4.22	21.66
H4	5.46	1.18	6.39	4.33	20.77
H5	5.38	1.24	6.46	4.18	22.88
H6	6.00	1.00	6.61	5.13	16.15
H7	5.82	1.07	6.53	4.83	18.08
H8	5.64	1.16	6.36	4.79	14.39
H9	5.69	1.00	6.45	4.76	19.10
H10	5.79	1.06	6.57	4.73	20.46
H11	5.57	1.16	6.35	4.59	16.34
H12	5.73	1.11	6.42	5.09	11.69
H13	6.00	0.92	6.53	5.28	13.95
H14	5.46	1.22	6.25	4.40	16.68
H15	5.26	1.32	6.15	4.17	16.84
H16	4.90	1.39	5.66	4.13	11.24
H17	5.07	1.32	5.83	4.44	10.81
H18	3.95	1.57	4.76	3.31	9.36
H19	5.09	1.16	5.75	4.55	10.29
H20	5.21	1.18	5.85	4.81	8.92
H21	5.16	1.17	5.93	4.39	14.36

Note. All the *t* values are statistically significant at the level of 0.05.

Table S2

The matrix of factor loadings for 21 items relating to the four factors extracted with an eigenvalue greater than 1 during principal component analysis

Item	Factor 1	Factor 2	Factor 3	Factor 4
H1	0.75	0.21	0.11	0.01
H2	0.79	0.28	0.15	< 0.01
H3	0.76	0.24	0.24	0.07
H4	0.76	0.31	0.18	0.13
H5	0.80	0.22	0.21	0.14
H6	0.36	0.68	0.20	0.06
H7	0.37	0.64	0.23	0.17
H8	0.16	0.29	0.18	0.35
H9	0.29	0.61	0.27	0.23
H10	0.34	0.55	0.32	0.19
H11	0.12	0.21	0.60	0.20
H12	0.18	0.29	0.28	0.21
H13	0.22	0.21	0.41	0.31
H14	0.18	0.13	0.86	0.10
H15	0.18	0.18	0.79	0.09
H16	0.11	0.13	0.36	0.10
H17	0.08	0.08	0.11	0.60
H18	0.22	0.02	0.12	0.20
H19	0.07	0.08	0.08	0.76
H20	-0.14	0.06	0.13	0.72
H21	0.14	0.19	0.14	0.70
**Accumulative variance explained**	36.04%	48.14%	55.90%	61.13%

Table S3

The matrix of factor loadings for the final 17 items by the factors of energy, tenacity, serenity and acuteness

Item	Energy	Tenacity	Serenity	Acuteness
H1. I feel alive.	0.76			
H2. I have a zest for life.	0.79			
H3. I am energetic.	0.77			
H4. I do things with great passion.	0.77			
H5. I feel I am full of energy.	0.81			
H6. I am a hard-working person.		0.66		
H7. I absorb new knowledge and improve my skills constantly.		0.66		
H9. I set goals firmly and put them into practice.		0.61		
H10. I don't give up easily.		0.54		
H11. I have a mild temper.			0.58	
H13. I am friendly to other people.			0.40	
H14. I consider myself emotionally stable.			0.88	
H15. I don't get mad easily.			0.78	
H17. I am interested in people.				0.59
H19. I am able to perceive inner changes in myself and others.				0.77
H20. I'm sensitive to changes in surrounding environment.				0.72
H21. On some occasions, I can quickly catch the changes in the situation.				0.70
**Accumulative variance explained**	40.64%	55.09%	63.96%	69.66%

Table S4

Correlations between FFVS and subjective vitality as measured with SVS

	Vitality (total score)	Energy	Tenacity	Serenity	Acuteness
Subjective vitality	0.75***	0.82***	0.62***	0.46***	0.24***

* $p < 0.05$; ** $p < 0.01$; *** $p < 0.001$.

Table S5

Correlations between FFVS and courage and its factors

	Vitality (total score)	Energy	Tenacity	Serenity	Acuteness
Courage	.78***	.70***	.75***	.53***	.46***
Bravery	.66***	.63***	.62***	.47***	.34***
Persistence	.70***	.59***	.74***	.45***	.42***
Authenticity	.66***	.50***	.60***	.52***	.49***
Zest	.77***	.77***	.72***	.48***	.41***

*** $p < .001$.

Table S6

Correlations between FFVS and well-being related variables

	Vitality (total score)	Energy	Tenacity	Serenity	Acuteness
Life satisfaction	.48***	.54***	.32***	.35***	.23***
Positive mental health	.63***	.63***	.50***	.49***	.32***
Resilience	.53***	.52***	.45***	.42***	.25***
Depression	-.43***	-.46***	-.33***	-.35***	-.16***
Loneliness	-.50***	-.52***	-.37***	-.42***	-.23***

** $p < .01$; *** $p < .001$.

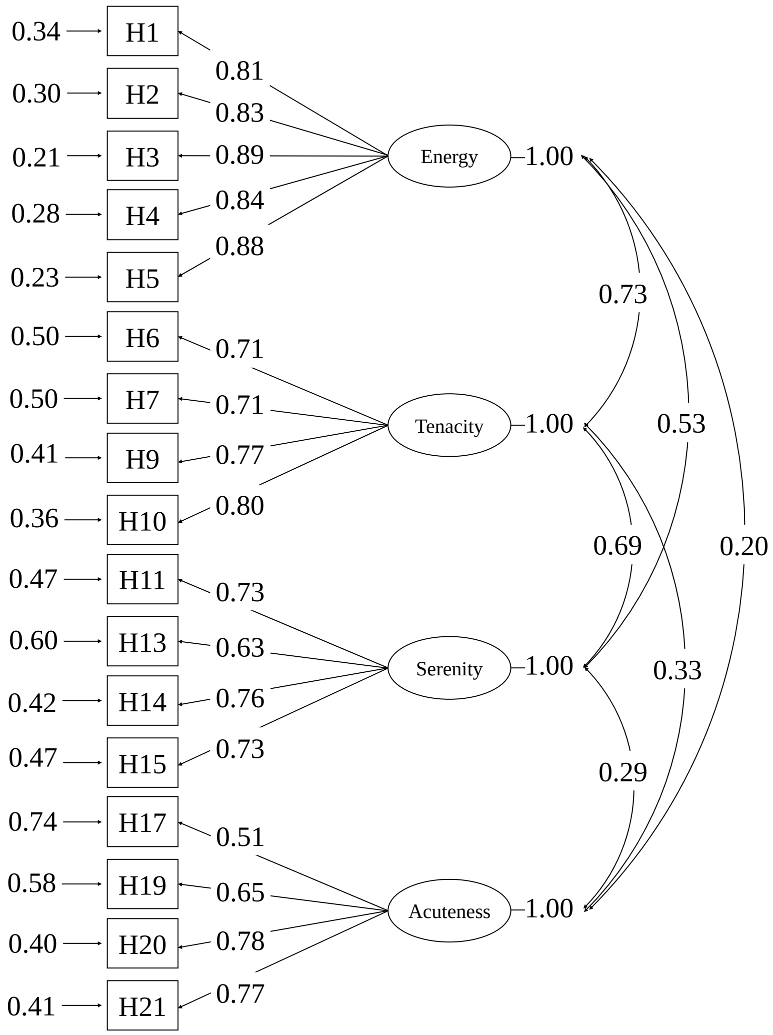

Figure S1 Results of CFA for the final 17 items

Attachment

Four-Factor Vitality Scale (FFVS; English translation)

Instructions:
The following items describe how you feel about your physical and mental energy and how you might harness them. Please read each item carefully and choose a number to represent your degree of agreement/disagreement with the item.

1	2	3	4	5	6	7
Strongly Disagree	Disagree	Slightly Disagree	Neutral	Slightly Agree	Agree	Strongly Agree

Energy
1. I feel alive.
2. I have a zest for life.
3. I am energetic.
4. I do things with great passion.
5. I feel I am full of energy.

Tenacity
6. I am a hard-working person.
7. I absorb new knowledge and improve my skills constantly.
8. I set goals firmly and put them into practice.
9. I don't give up easily.

Serenity
10. I have a mild temper.
11. I am friendly to other people.
12. I consider myself emotionally stable.
13. I don't get mad easily.

Acuteness
14. I am interested in people.
15. I am able to perceive inner changes in myself and others.
16. I'm sensitive to changes in surrounding environment.
17. On some occasions, I can quickly catch the changes in the situation.

Scoring:
The general level of vitality is obtained by summing up all the item scores, and the level of each vitality factor is obtained by summing up the score of items for each factor.

四维度活力量表（Original Chinese Version）

指导语：
下列陈述描述了你对自身精神能量和生理能量的体验，以及你如何使用这些能量。请仔细阅读每一条陈述，并选择一个数字表明你对陈述的同意或不同意程度。

1	2	3	4	5	6	7
非常<br>不同意	不同意	有些<br>不同意	中立	有些同意	同意	非常同意

<u>**能量**</u>
1. 我感到充满朝气。
2. 我对生活充满热情。
3. 我精力充沛。
4. 我做事充满激情。
5. 我感觉自己充满力量。

<u>**坚韧**</u>
6. 我是个努力工作的人。
7. 我喜欢不断学习新的知识和提升我的技能。
8. 我坚定目标，同时付诸实践。
9. 我不轻易放弃。

<u>**平和**</u>
10. 我脾气温和。
11. 我待人和气。
12. 我情绪比较稳定。
13. 我不轻易生气。

<u>**敏锐**</u>
14. 我爱观察他人。
15. 我能察觉到自己和别人的内心变化。
16. 我对周围环境变化比较敏感。
17. 在一些场合，我可以快速捕捉到情况的变化。

计分说明：
将所有陈述的分数加和得到量表总分，代表四维度活力的总体水平；将各维度陈述的分数分别加和得到维度总分，代表四维度活力的维度水平。
